# Predicting antimicrobial resistance of bacterial pathogens using time series analysis

**DOI:** 10.3389/fmicb.2023.1160224

**Published:** 2023-05-11

**Authors:** Jeonghoon Kim, Ruwini Rupasinghe, Avishai Halev, Chao Huang, Shahbaz Rezaei, Maria J. Clavijo, Rebecca C. Robbins, Beatriz Martínez-López, Xin Liu

**Affiliations:** ^1^Department of Mathematics, University of California, Davis, Davis, CA, United States; ^2^Department of Medicine and Epidemiology, Center for Animal Disease Modeling and Surveillance (CADMS), School of Veterinary Medicine, University of California, Davis, Davis, CA, United States; ^3^Department of Computer Science, University of California, Davis, Davis, CA, United States; ^4^Department of Veterinary Diagnostic & Production Animal Medicine (VDPAM), Iowa State University, Ames, IA, United States; ^5^R.C. Robbins Swine Consulting Services, PLLC, Amarillo, TX, United States

**Keywords:** antimicrobial resistance, antibiotics, bacterial pathogen, time series analysis, SARIMA

## Abstract

Antimicrobial resistance (AMR) is arguably one of the major health and economic challenges in our society. A key aspect of tackling AMR is rapid and accurate detection of the emergence and spread of AMR in food animal production, which requires routine AMR surveillance. However, AMR detection can be expensive and time-consuming considering the growth rate of the bacteria and the most commonly used analytical procedures, such as Minimum Inhibitory Concentration (MIC) testing. To mitigate this issue, we utilized machine learning to predict the future AMR burden of bacterial pathogens. We collected pathogen and antimicrobial data from >600 farms in the United States from 2010 to 2021 to generate AMR time series data. Our prediction focused on five bacterial pathogens (*Escherichia coli, Streptococcus suis, Salmonella sp., Pasteurella multocida*, and *Bordetella bronchiseptica*). We found that Seasonal Auto-Regressive Integrated Moving Average (SARIMA) outperformed five baselines, including Auto-Regressive Moving Average (ARMA) and Auto-Regressive Integrated Moving Average (ARIMA). We hope this study provides valuable tools to predict the AMR burden not only of the pathogens assessed in this study but also of other bacterial pathogens.

## 1. Introduction

The discovery of antimicrobials is one of the best advances in therapeutic medicine in humans and animals. Over time, microbes have evolved and developed resistance mechanisms against these antimicrobial compounds. Increasing resistance to the available antimicrobials and stagnation of developing novel antimicrobials limit treatment options for patients with infectious diseases. Therefore, the emergence, dissemination, and persistence of microbes that are resistant to existing antimicrobials pose an enormous threat to public and animal health. Antimicrobials are extensively used in the food animal industry to treat bacterial infections and promote health, welfare, and production. According to Food and Drug Administration (FDA), ~80% of all antibiotics in the United States in 2011 were sold for use in animal husbandry, and ~70% of them belonged to the antibiotic classes used in human medicine (medically important antibiotics; FDA Department of Health and Human Services, 2011). Pig farming is one of the leading sectors using antimicrobials. Thus, increased levels of AMR are anticipated in swine farms due to the selective pressure of these antimicrobials and can spread via pork, direct contact with pigs, or discharge of swine waste into the environment.

A key to preventing AMR emergence and spread is early and accurate detection of potential AMR, which promotes selecting appropriate antimicrobials and facilitating the prompt investigation of drug-resistant disease outbreaks. Routine monitoring and surveillance can enable exemplary stewardship by detecting AMR emergence, tracing AMR patterns, and effectively targeting antimicrobial interventions and mitigation strategies. Currently, antimicrobial susceptibility testing (AST) is the primary method for detecting AMR and selecting effective antimicrobials against bacterial pathogens, which involves culturing the bacteria in the presence of a panel of various antimicrobials. Effective antimicrobials can be determined by detecting Minimum Inhibitory Concentration (MIC), where antimicrobials with lower MIC values are considered more effective (susceptible) because less of the drug is needed to inhibit bacterial growth. However, these procedures can be expensive and time-consuming, depending on the growth rate of the bacteria and MIC testing procedures. Alternative methods, such as DNA sequencing technologies, are increasingly used to detect AMR at the molecular level, but they require robust bioinformatics tools to evaluate the genomic structure of the microbial resistomes. Thus, most clinical laboratories still depend primarily on conventional AST to conduct clinical therapy and observe AMR over time. Nevertheless, most farms may not have the resources (e.g., time and budget) to perform routine testing to detect AMR and quantify the AMR burden in field settings. Therefore, developing a tool to predict AMR burden based on available data, such as prior AMR information (susceptible/resistance) against common antimicrobials, could be very useful to better inform decision-making about antimicrobial use at the farm level, which consequently helps mitigate AMR.

Machine learning has been widely employed for studying AMR, highlighting its importance in predicting resistance levels mainly using features directly from genotypes (Pesesky et al., 2016; Nguyen et al., 2018, 2019; Wang et al., 2022). However, there are situations where we do not obtain genomic data to predict AMR levels but only preserve historical phenotype information. Time series analysis is a great solution to relevant tasks for such situations. Time series has shown great performances in studying AMR (López-Lozano et al., 2000; Hsueh et al., 2005; Aldeyab et al., 2008; Guo et al., 2019; Jeffrey et al., 2021; Strahlberg, 2021), and sometimes their methods are limited to Auto-regressive Integrated Moving Average (ARIMA; Chatfield, 2003) or subcategory methods that cannot properly incorporate seasonal behavior of AMR levels. Among many time series approaches, the Seasonal Auto-regressive Integrated Moving Average (SARIMA; Chatfield, 2003) has received significant attention because of its outstanding performance in time series forecasting. SARIMA shows its usefulness when some degrees of seasonality-periodic fluctuations occur repeatedly in the time series.

In this study, we used the SARIMA algorithm to predict the future burden of AMR (AMR proportions) of five bacterial pathogens (*Escherichia coli, Streptococcus suis, Salmonella sp., Pasteurella multocida*, and *Bordetella bronchiseptica*) prevalent in the studied swine farms using the prior resistance information. The data included the number of tested pathogens with confirmed resistance (based on MIC interpretations) to their corresponding antimicrobials. Instead of direct use of binary (susceptible and resistance) classified data, we generated integrated time series data, i.e., quarterly-based AMR proportions for each of the study pathogens. This approach enabled us to overcome the limitations of missing data over time. We also compared the performance of SARIMA to that of Auto-Regressive Moving Average (ARMA; Wold, 1938), Auto-Regressive Integrated Moving Average (ARIMA; Chatfield, 2003), and three other forecasting baseline methods as follows: Naïve, Seasonal Naïve, and one-lagged prediction (Ryu and Sanchez, 2003; Reza Hoshmand, 2009). These three baselines were selected as benchmarks in our study because they are often used in forecasting tasks and are simple yet effective. We believe that predicting AMR proportions using time series models can provide valuable information to facilitate the selection of appropriate antimicrobials against pathogens and the prompt investigation of drug-resistance disease outbreaks.

## 2. Materials and methods

**Figure 1:**
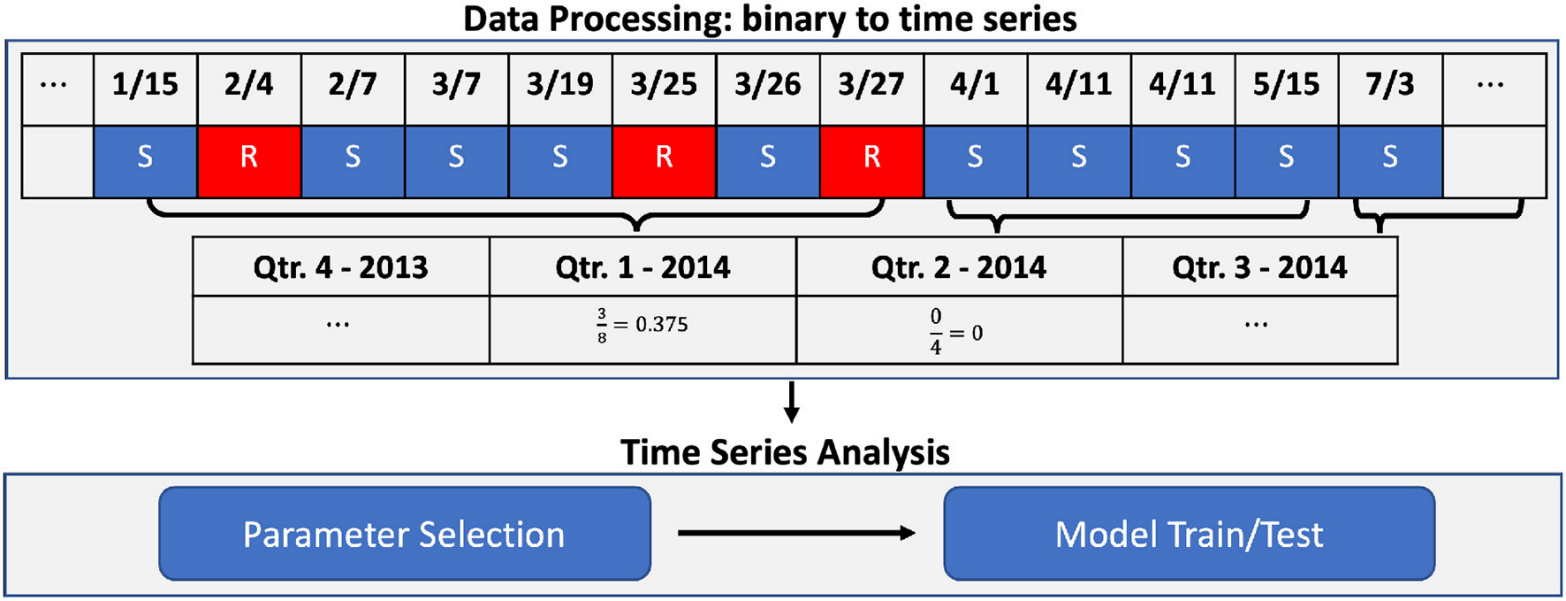
Workflow chart. Data processing example [from irregular binary data to quarterly based time series: *Res*(*Pathogen, Antimicrobial*)] and time series analysis.

In this section, we discuss the workflow, time series analysis methods, and experimental design. Workflow after data collection includes data processing (irregular binary data to quarterly time series data) and time series analysis (model parameter selection and model train/test; Figure 1).

### 2.1. Data collection

In this study, we used pathogen and antimicrobial information from >600 farms owned by two swine production systems in the United States. The samples were collected from pigs infected with one of five bacterial pathogens (*Escherichia coli, Streptococcus suis, Salmonella sp., Pasteurella multocida*, and *Bordetella bronchiseptica*) from 2010 to 2021 and tested for AMR against a panel of antimicrobials (Table 1). The resistance level of each pathogen against antimicrobials was detected by determining MIC and classified into two groups as follows: susceptible (*S*) and resistant (*R*), based on an interpretation report received from the American Association of Veterinary Laboratory Diagnosticians (AAVLD) accredited laboratory in the United States.

**Table 1:** Full data of antimicrobial and pathogens used for study.

	***Escherichia coli***	***Streptococcus suis***	***Salmonella sp*.**	***Pasteurella multocida***	***Bordetella bronchiseptica***
Clindamycin	1.0/0.0	0.8/0.18	1.0/0.0	1.0/0.01	1.0/0.0
Tiamulin	0.99/0.03	0.16/0.1	1.0/0.01	0.58/0.29	1.0/0.01
Tylosin	–	–	–	0.98/0.06	–
Ampicillin	0.71/0.22	0.03/0.06	0.58/0.27	0.03/0.07	1.0/0.02
Gentamicin	0.32/0.16	–	0.51/0.39	–	0.04/0.1
Oxytetracycline	0.88/0.13	0.93/0.1	–	0.23/0.26	0.03/0.1
Penicillin	1.0/0.0	0.18/0.13	1.0/0.0	0.19/0.28	1.0/0.0
Spectinomycin	0.9/0.22	–	–	–	–
Tilmicosin	0.99/0.03	0.73/0.21	1.0/0.0	0.21/0.31	–
Chlortetracycline	0.88/0.13	0.93/0.1	–	0.03/0.07	0.04/0.11
Sulphadimethoxine	–	0.61/0.22	–	–	0.97/0.08
Ceftiofur	–	0.04/0.06	–	0.0/0.02	–
Enrofloxacin	0.34/0.25	0.07/0.07	0.29/0.38	0.0/0.02	–
Florfenicol	0.84/0.09	0.03/0.07	0.9/0.14	0.02/0.07	0.83/0.17
Neomycin	0.34/0.25	0.73/0.17	0.57/0.39	0.07/0.14	–
Sulfa./trimethoprim	0.26/0.27	–	0.32/0.32	–	0.89/0.14
Tulathromycin	–	–	–	0.0/0.01	0.04/0.1

Note that – indicates that corresponding data are not used in the experiments.

Two numbers provided indicate mean and standard deviation of data, i.e., an indicator for averaged AMR proportions and how dynamically (uncertain) the time series is changing, respectively.

### 2.2. Data processing for time series analysis

**Figure 2:**
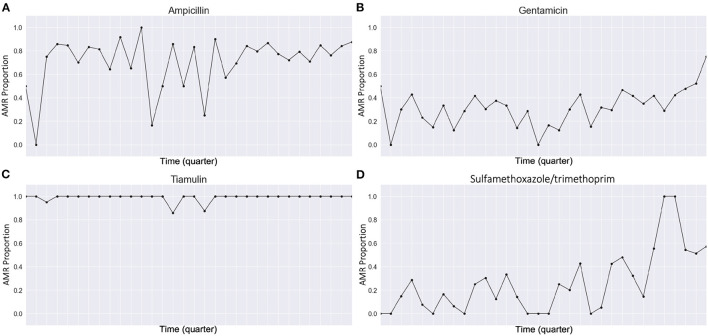
Examples of our processed quarterly based AMR proportions time series. **(A)** *Res(Escherichia coli, Ampicillin)*, **(B)** *Res(Escherichia coli, Gentamicin)*, **(C)** *Res(Escherichia coli, Tiamulin)*, and **(D)** *Res(Escherichia coli, Sulfamethoxazole/trimethoprim)*.

For each pathogen, different groups of antimicrobials were employed for experiments (see Table 1). One challenge is that there were missing data points between certain time periods. To tackle this, we constructed a quarterly time series dataset by integrating the data points every quarter. We converted our data points to a quarterly basis dataset and define *Res*(*Pathogen, Antimicrobial*) the resistance time series for each pathogen and antimicrobial as below.


(1) $$\begin{array}{c} Res\left(Pathogen,Antimicrobial\right)=\left(r_{1},r_{2},\cdots \;,r_{n}\right), \end{array}$$

where $r_{i}=Proportion\left(R\right)=\frac{\#\text{of}R}{\#\text{of (}R\text{+}S\text{)}}$ over the *i*th quarter (*R* and *S* stand for resistant and susceptible, respectively). Figure 1 shows how we processed our dataset. Figure 2 shows examples of quarterly based time series constructed for pathogens and antimicrobials, and all of the time series examples are presented as solid lines in Supplementary Figures 1–5. With the constructed data, we focused on predicting AMR proportions in times series for each *Res*(*Pathogen, Antimicrobial*) in our data.

We also output the mean and the standard deviations of *Res*(*Pathogen, Antimicrobial*) which can be an indicator for the averaged AMR proportions and dynamics of each time series (Table 1). We also observed different degrees of fluctuation in the processed dataset. For example, *Res*(*Escherichia coli, Ampicillin*) changes more dynamically than *Res*(*Escherichia coli, Tiamulin*) (Figure 2 and Table 1).

### 2.3. AR(I)MA, SARIMA, and three baselines

#### 2.3.1. ARMA and ARIMA

Auto-Regressive Moving Average (ARMA) model consists of two parts, such as autoregressive (AR) and moving average (MA) parts (Wold, 1938). The model is usually referred to as *ARMA*(*p, q*), where *p* and *q* are the order of the AR and MA parts, respectively (Valipour et al., 2012). AR part takes previous observations as inputs to predict future values. MA part uses previous errors between predicted and observed as predictors for future values. *ARIMA* model consists of three parts, such as AR, MA, and the integrated (I) parts (Chatfield, 2003). The model is usually referred to as *ARIMA*(*p, d, q*), where *p* and *q* are the same as for the ARMA model, and *d* is the degree of differencing. The integrated part refers to the differencing of observations to allow time series to become stationary.

#### 2.3.2. SARIMA

Seasonal Auto-regressive Integrated Moving Average (SARIMA) model (Chatfield, 2003), as an advanced method of ARIMA with a seasonal component, overcomes the limitation that ARIMA cannot tackle data with periodic behavior properly. In this study, we employed SARIMA to predict AMR proportions for bacterial pathogens considering AMR proportions vary over time with a potential seasonality.

A typical SARIMA model has seven parameters, referred to as *SARIMA*(*p, d, q*)(*P, D, Q*)_*S*_, where (*p, q*) and (*P, Q*) are the order of the non-seasonal and seasonal (autoregressive, moving) models, respectively, *d* and *D* are the numbers of non-seasonal and seasonal differences, respectively, and *S* is the periodic seasonality term. Choosing appropriate parameters is a key process for the optimal SARIMA performance. To this end, autocorrelation and partial autocorrelation functions are utilized. To be precise, we first determine non-seasonality components (*p, d, q*), and then, we find proper seasonal parameters (*P, D, Q*)_*S*_ using autocorrelation function and partial autocorrelation function. Time series datasets often have trends in time series and changes in the statistical structure of the series, which means non-stationarity. To find non-seasonality parameters, trend and seasonality in time series should be removed using differencing techniques. After the removal of trend and seasonality, the autocorrelation function and partial autocorrelation function help determine non-seasonal parameters. Additionally, we also check the *p*-value between time series data and its lagged time series, and the number of lags with the lowest *p*-value determines seasonality parameter S for the SARIMA model. However, these steps do not always guarantee finding a specific set of parameters for the optimal SARIMA model. In many cases, parameter exploration using grid search is required, which means that we set some possible candidates for parameters and check the SARIMA model performance to find sets of parameters with the best performance.

#### 2.3.3. Three baselines

*Naïve* method is the simplest time series forecasting method where all remaining forecast is set equal to the observation made in the last timestamp as below.


(2) $$\begin{array}{c} F_{T+t}=Y_{T}\text{for}t>0, \end{array}$$

where *F* and *Y* are forecasting and observed times series, respectively. *T* and *T*+*t* are the timestamps of the last observation and the forecast time, respectively.

*Seasonal Naïve* method is an extension of the Naïve method with a seasonality. It predicts the forecasts based on the same timestamp in the previous cycle as below.


(3) $$\begin{array}{c} F_{T+t}=Y_{T+t-s\left(k-1\right)}\text{for}t>0, \end{array}$$

where *s* is seasonality and *k* is completed cycles.

*One-lagged prediction* methods rely on the most recently acquired data (Ryu and Sanchez, 2003). One-lagged prediction utilizes the data from the previous timestamp to forecast the current timestamp as shown below.


(4) $$\begin{array}{c} F_{T+1}=Y_{T}\text{for}t>0. \end{array}$$

### 2.4. Experiments

#### 2.4.1. Parameter selection for SARIMA

For accurate AMR time series prediction, it is crucial to find appropriate SARIMA parameters (Ma et al., 2021). We selected *Escherichia coli* and *Neomycin* because *Res*(*Escherichia coli, Neomycin*) provides the largest number of data points to work with, and it shows visible seasonality. We have seven parameters to determine as follows: (*p, d, q*), (*P, D, Q*), and *S*. After using the differencing method to find parameter *d* and to remove the trend component in *Res*(*Pathogen, Antimicrobial*), autocorrelation function, partial autocorrelation function, *p*-value analysis, and parameter exploration were attempted to assessing SARIMA parameters. We choose optimal SARIMA parameters that predict *Res*(*Escherichia coli, Neomycin*) with the lowest error.

#### 2.4.2. ARMA and ARIMA parameter selection

Similar to SARIMA, we also employed parameter exploration to find the optimal parameters for ARMA and ARIMA. *Res*(*Escherichia coli, Neomycin*) was utilized for this process. We conducted two experiments for ARMA and ARIMA independently because ARMA does not take parameter *d* into account while ARIMA considers it.

#### 2.4.3. Time series-based AMR proportions prediction

We selected seven combinations of parameters from previous analysis on *Res*(*Escherichia coli, Neomycin*) and applied the chosen seven combinations of parameters to other *Res*(*Pathogen, Antimicrobial*) to predict the AMR proportions. Specifically, for each *Res*(*Pathogen, Antimicrobial*), seven experiments with different parameter sets were conducted. Each experiment returned a rooted mean squared error as a performance measurement. We also used three baselines as follows: Naïve, Seasonal Naïve (we set four as the seasonality period), and one-lagged prediction. All baselines also outputted root mean squared error values for each *Res*(*Pathogen, Antimicrobial*). All experiments were conducted in Python (version 3.7.6).

## 3. Results

### 3.1. Seven selected sets of SARIMA parameters

**Figure 3:**
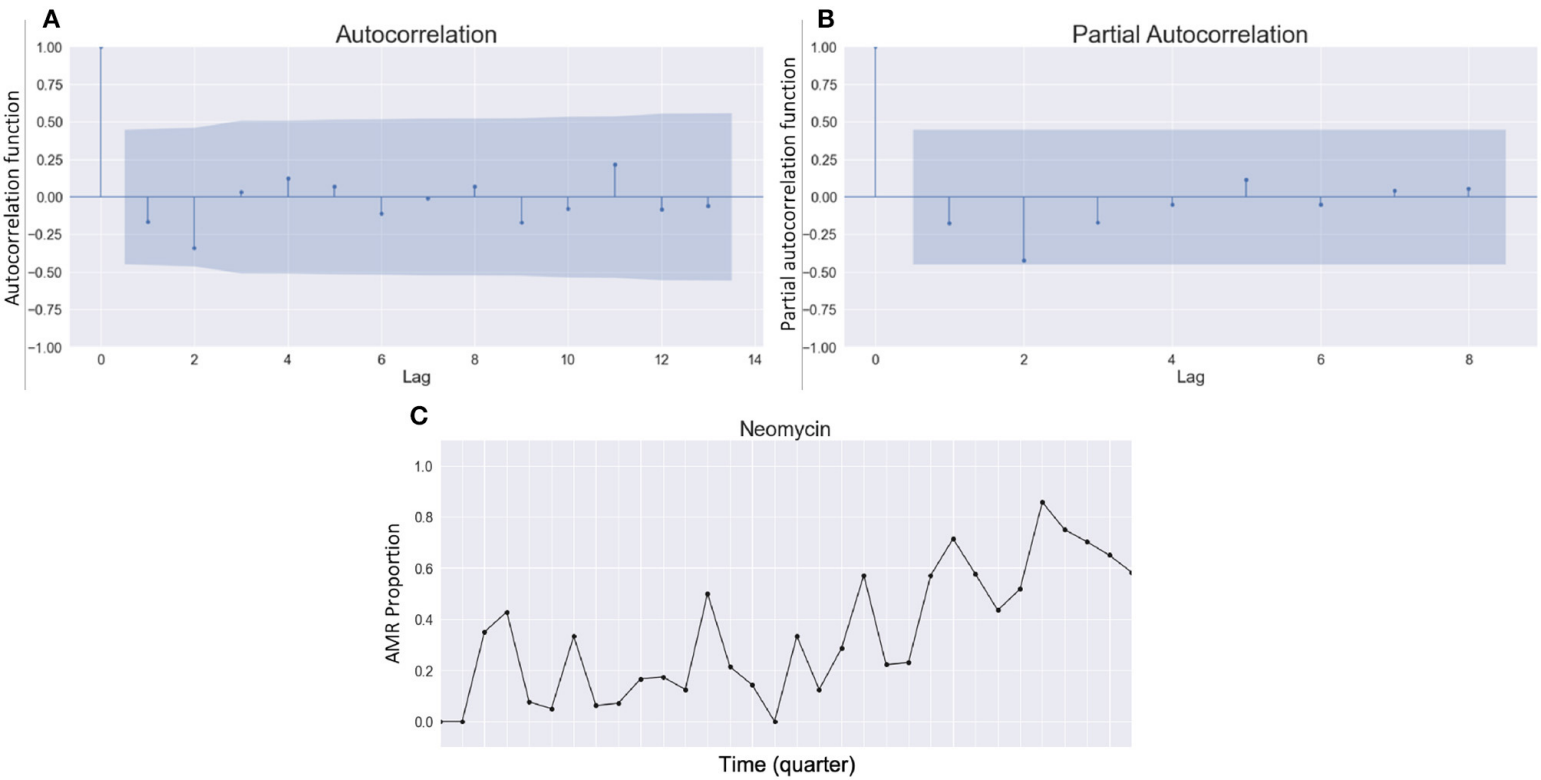
Autocorrelation function and partial autocorrelation function analysis for parameter selection. **(A)** Autocorrelation function plot, **(B)** partial autocorrelation function plot, and **(C)** AMR time series for *Escherichia coli* and Neomycin, i.e., *Res(Escherichia coli, Neomycin)*.

As shown in Figure 3, the autocorrelation function and partial autocorrelation function provided information on choosing the right parameters for SARIMA. *P*-value analysis for the *Res*(*Escherichia coli, Neomycin*) and its lagged time series with a different number of lags were also used to find the seasonal parameter S. From these, we can determine our parameter S = 12, but other parameters were not found properly from autocorrelation function and partial autocorrelation function analysis. There were no significant patterns of gradual decay or recurring cycles observed in either the autocorrelation or partial autocorrelation plots (Figure 3). Specifically, there is no data point with a lag value greater than zero that fell outside the confidence interval (blue shade area) in either plot (Figure 3), resulting in making it unable to estimate the appropriate parameters for a moving average (MA) or autoregressive (AR) models. From these analyses, the parameters of the time series model could not be satisfactorily determined without a parameter search.

In this regard, we explore the set of parameters that output the lowest error estimation measured by rooted mean squared error. In other words, we conducted trial and error for finding appropriate undetermined parameters remained. Our parameter exploration includes integers from 0 to 5 for three parameters *p*, *d*, *q*, and from 0 to 6 for the other three parameters *P*, *D*, *Q*, for which we end up having 6^3^7^3^ combinations to attempt. For each attempt with a combination of parameters, SARIMA predicted *Res*(*Escherichia coli, Neomycin*), i.e., tried to predict the 10% of the last values in *Res*(*Escherichiacoli, Neomycin*) after being trained with the first 90% of the *Res*(*Escherichia coli, Neomycin*), and a prediction error was reported. With outputted errors, we selected seven parameter combinations that return the lowest rooted mean squared error values because the next best one after these seven has a relatively big gap in the errors from the first seven parameters, and interestingly, our three parameters (*P, D, Q*)_*S*_ are fixed as (1, 0, 1)_12_ while seven different (*p, d, q*) are acquired from Table 2.

**Table 2:** Seven selected parameters of SARIMA, ARMA, and ARIMA used for overall AMR proportions prediction acquired from *Res(Escherichia coli, Neomycin)* analysis.

	**SARIMA**							**ARMA**			**ARIMA**			
**No**.	**p**	**d**	**q**	**P**	**D**	**Q**	**S**	**No**.	**p**	**q**	**No**.	**p**	**d**	**q**
#1	1	1	4	1	0	1	12	#1	3	2	#1	3	2	1
#2	2	2	3	1	0	1	12	#2	3	0	#2	2	2	3
#3	3	0	4	1	0	1	12	#3	3	1	#3	2	2	1
#4	3	1	4	1	0	1	12	#4	1	0	#4	3	2	2
#5	4	0	0	1	0	1	12	#5	1	1	#5	3	1	0
#6	4	3	4	1	0	1	12	#6	1	2	#6	2	1	0
#7	4	2	4	1	0	1	12	#7	1	3	#7	2	2	2

### 3.2. Seven parameter sets for ARMA and ARIMA

To find the parameter sets that predicted *Res*(*Escherichia coli, Neomycin*) with the lowest errors, we explored integers from 0 to 5 for all parameters (*p, q*) and (*p, d, q*) for ARMA and ARIMA, respectively. Each experiment requires 6^3^ and 6^2^ iterations to search independently. In the end, seven sets of (*p, d, q*) and (*p, q*) that outputted the lowest rooted mean squared error were selected (Table 2).

### 3.3. Error estimation for AMR proportions prediction

**Figure 4:**
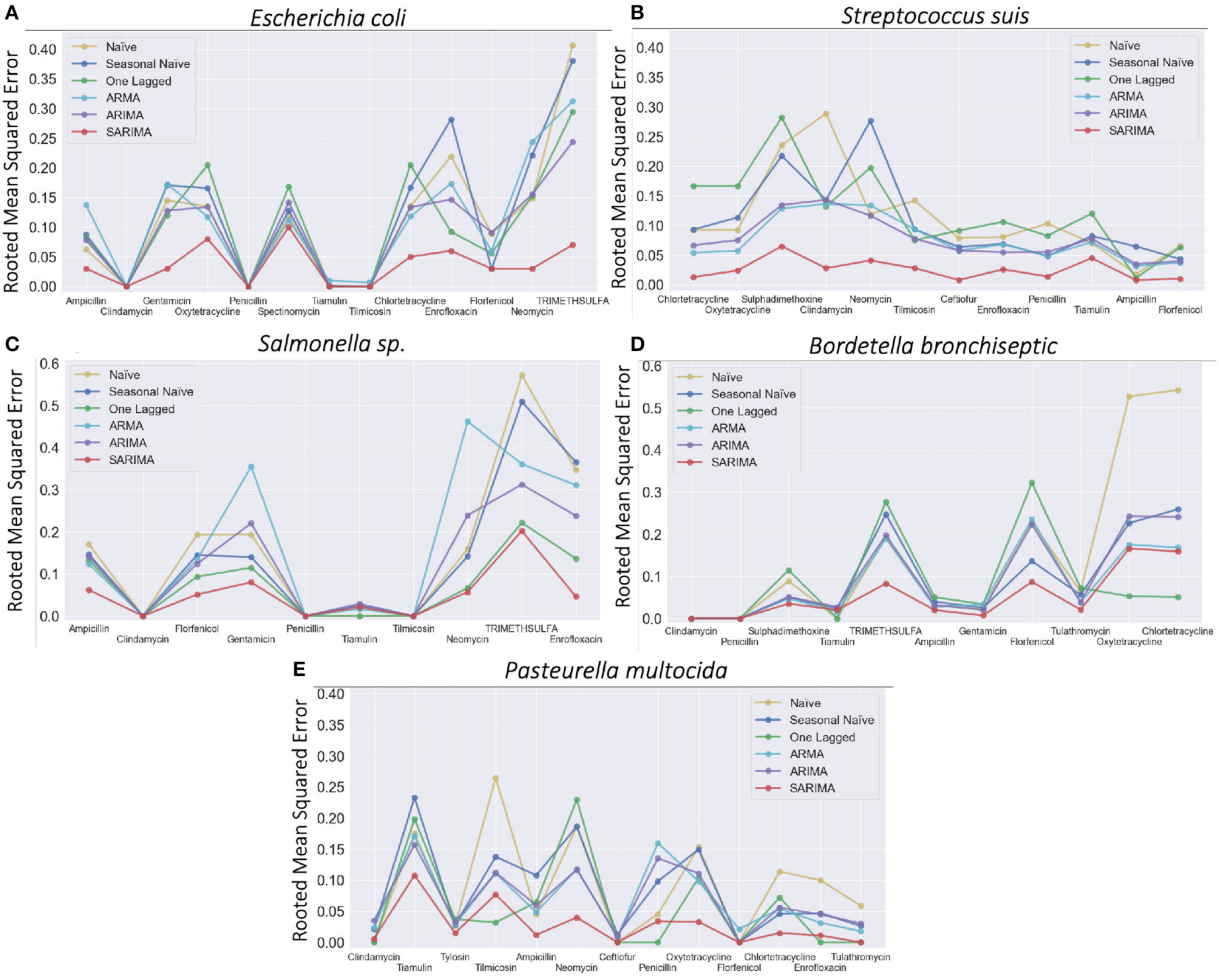
Rooted mean squared errors for five pathogens with corresponding antimicrobials. **(A)** *Escherichia coli*, **(B)** *Streptococcus suis*, **(C)** *Salmonella sp*., **(D)** *Bordetella bronchiseptica*, and **(E)** *Pasteurella multocida*.

For each *Res(Pathogen, Antimicrobial)* time series prediction using SARIMA, the seven previously selected SARIMA parameter sets were applied. Each experiment outputted a rooted mean squared error value which represents how good the prediction is, i.e., the lower the rooted mean squared error value, the more accurate the method (Figure 4). The lowest error value was provided among seven errors from seven experiments of SARIMA for each *Res(Pathogen, Antimicrobial)*. For each ARMA and ARIMA, seven parameters were conducted, and the lowest rooted mean squared error values were outputted among seven different experiments. We observed that our SARIMA method showed lower rooted mean squared error values compared to ARMA, ARIMA, and the other three baselines in general. The rooted mean squared error gap between SARIMA and three baselines became bigger when the AMR proportion time series [*Res*(*Pathogen, Antimicrobial*)] have greater deviation values (equivalently, more dynamical). This is because higher deviation implies more fluctuation in AMR proportion time series that are harder to predict. For example, rooted mean squared error values were similar between SARIMA and three baselines for *Res*(*Escherichia coli, Tilmicosin*) (standard deviation: 0.03), while root mean squared error gap became bigger for *Res*(*Escherichia coli, Enrofloxacin*) (standard deviation: 0.03; Figure 4 and Table 1).

## 4. Discussion

This study investigated the plausibility of executing data-driven forecasting of the future AMR burden using the available resistance data in >600 swine farms in the United States from 2010 to 2021. AMR burden was quantified quarterly by calculating the proportions of resistant strains of five crucial bacterial pathogens (*Escherichia coli, Streptococcus suis, Salmonella sp., Pasteurella multocida*, and *Bordetella bronchiseptica*) against their corresponding antimicrobials. The bacterial species assessed in this study were the most prevalent swine bacterial pathogens dispersed within the studied farms, significantly affecting their health, welfare, and productivity. These pathogens can cause various infections in pigs, including respiratory, gastrointestinal, and/or systemic infections, and antimicrobials are the primary mode of therapy and prevention of these infectious diseases (Robbins et al., 2014). Therefore, early and accurate detection of potential AMR of these pathogens is essential to determine the appropriate antimicrobials to use against and monitor for drug-resistant disease outbreaks. In this study, we used three machine learning-based time series analyses to predict the future AMR proportions in the studied farms and compared their performances to select the most efficient and accurate approach for future use. According to our findings, SARIMA predicted AMR proportions accurately and outperformed ARMA, ARIMA, and three baselines according to the rooted mean squared error value. However, parameter exploration remains a light limitation due to the potential computational burden because the key to prediction using SARIMA was to find appropriate parameters which cannot always be acquired from the general process using partial autocorrelation function.

According to this study, we observed distinct temporal trends in AMR proportions for the five pathogens against their corresponding antimicrobials during the study period (Supplementary Figures 1–5). For example, pathogens, such as *Escherichia coli* and *Salmonella sp*., showed very high or increasing trends of AMR proportions against Enrofloxacin, Neomycin, Sulfamethoxazole/trimethoprim, and Clindamycin, etc., while *Streptococcus suis* exhibited low resistance to ampicillin, ceftiofur, enrofloxacin, florfenicol, and tiamulin. Most of the studied antimicrobials are effective against *Pasteurella multocida*, whereas *Bordetella bronchiseptica* displayed higher resistance levels against most antimicrobials assessed in our study. Nevertheless, these quarterly based-AMR proportions showed frequent fluctuations in most pathogens against their corresponding antimicrobials throughout the study period (Supplementary Figures 1–5). However, our SARIMA models were able to correctly capture all these individual trends and predict the future AMR proportions with high accuracy. Specifically, our study demonstrated that SARIMA works well for dynamic time series, such as AMR proportion time series for the studied five pathogens even if it is difficult to fairly compare our results to those from other relevant studies, as each system has its unique data samples and methods. In addition, this method could be applied to predict other unexplored pathogens unless the available data are limited. In other words, this work can be generalized to AMR proportion time series for any pairs of pathogens and antimicrobials. Furthermore, our SARIMA model can also be applied to other time series analyses in the domain, such as swine mortality rate.

Early detection of emerging AMR and future prediction of AMR burden and trends are vital to comprehend the extent of the threat and implement appropriate antimicrobial interventions and mitigation strategies. Numerous studies have explored various ML algorithms to study AMR using available phenotypic data (López-Lozano et al., 2000; Hsueh et al., 2005; Aldeyab et al., 2008; Guo et al., 2019; Jeffrey et al., 2021; Strahlberg, 2021) and genotypes (Pesesky et al., 2016; Nguyen et al., 2018, 2019; Wang et al., 2022). Specifically, the recent advancements in affordable and rapid DNA sequencing technologies (e.g., whole genome sequencing) combined with ML approaches have drastically transformed AMR surveillance and prediction prospects. Predicting pathogens that might express AMR by using their genomics data has shown great promise in the real-time detection of AMR determinants. However, this process requires robust bioinformatics tools and advanced analytical skillsets to assess the microbial genomic structure and the resistomes, and these limitations still preclude cost-effective, user-friendly, and rapid antimicrobial resistance surveillance. In addition, phenotyping approaches provide direct visual evidence of interaction between a bacterial strain and an antimicrobial. Thus, most clinical laboratories, to date, rely mainly on traditional AST to guide clinical therapy and monitor AMR over time. Therefore, the SARIMA model we proposed in our study will be an efficient and practical alternative to predict AMR burden, especially for situations where we do not have genomic data but only have historical phenotype information.

There are a few limitations to our study. The AMR data used for prediction were comprised of data from multiple swine farms within the United States. Although these farms were managed under two major swine production systems, individual farms can have different management practices, biosecurity measures, treatment protocols, etc. Previous studies disclosed various factors, such as transportation, farm management, housing conditions, metals consumption, feeding strategies, antimicrobial usage, and co-infections that can affect the spread of antimicrobial-resistant bacteria and the AMR levels in a farm (Mathew et al., 2003; Dewulf et al., 2007; Medardus et al., 2014; Luiken et al., 2022; Odland et al., 2022). However, we did not incorporate these factors in our study. Thus, the future AMR burden (proportions) can vary from the predicted levels due to the variations in these farm factors. Since the AMR predictions were made using a limited number of swine farms in the United States, we cannot generalize our findings to the entire swine population in the United States. Therefore, we cannot generalize our findings to the entire swine population in the United States. However, our results depict the potential of using time series analysis to predict AMR levels within a farm or geographical region. In this study, we transformed the AMR data into a binary variable (susceptible/resistance) using breakpoints acquired from the interpretation report from AAVLD-accredited laboratories in the United States. Some of these breakpoints were extrapolated from other species (e.g., human and canine) if swine-specific breakpoints were not available for a pathogen–antimicrobial combination (Watts et al., 2018; Lubbers et al., 2020). Breakpoint MICs depend on the clinical pharmacology of antimicrobials and are generally specific for bacterial-antimicrobial-host-disease-tissue-dosing regimen combinations (Watts et al., 2018; CLSI, 2019; Lubbers et al., 2020); thus, different testing laboratories may use different standards for resistance classifications, which may cause misclassifications of pathogens. Nevertheless, predicting AMR burden directly from MIC values will minimize these misclassifications or classification errors. Hence, future studies are suggested to perform time series analysis based on the raw MIC data.

## 5. Conclusion

This study proposed to use time series methods for the prediction of future AMR burden by constructing the quarterly based AMR proportion times series. The SARIMA approach showed low errors in terms of rooted mean squared error compared with ARMA, ARIMA, and three other forecasting baselines, and it worked even for highly dynamic time series. We believe that our time series prediction can help to advise using appropriate antimicrobials and reduce the risk related to AMR events by predicting anticipation of AMR occurrences in farms or geographical regions. Furthermore, our study may also contribute to the analysis of similar problems and scenarios.

## Data availability statement

The data analyzed in this study is subject to the following licenses/restrictions: The dataset used in this study cannot be publicly available. Requests to access these datasets should be directed to BM-L, beamartinezlopez@ucdavis.edu.

## Author contributions

JK designed the study, developed the Python codes for experiments, carried out the analysis of experimental results, and wrote the draft of the manuscript. BM-L and RR collected, cleaned, and verified the data. RR contributed to writing the introduction and discussion parts, especially from biological perspectives. AH, CH, SR, and XL helped to justify methodologies in this study and to edit the method explanation part. MC and RCR helped to develop ideas and analyze the results. All authors participated in the discussion and interpretation of the results and read, edited, and approved the final manuscript.
