# Supplementary material for: Predicting antimicrobial resistance of bacterial pathogens using time series analysis
Source: Front Microbiol. 2023 May 11;14:1160224. doi: 10.3389/fmicb.2023.1160224 (PMC10213968; doi:10.3389/fmicb.2023.1160224)

Supplementary 1. Prediction results on *E. Coli.* time series data samples based on the SARIMA (e.g. of 9 antibiotics). Solid and dotted lines are observed and predicted values, respectively, and the grey area is 95% prediction intervals.

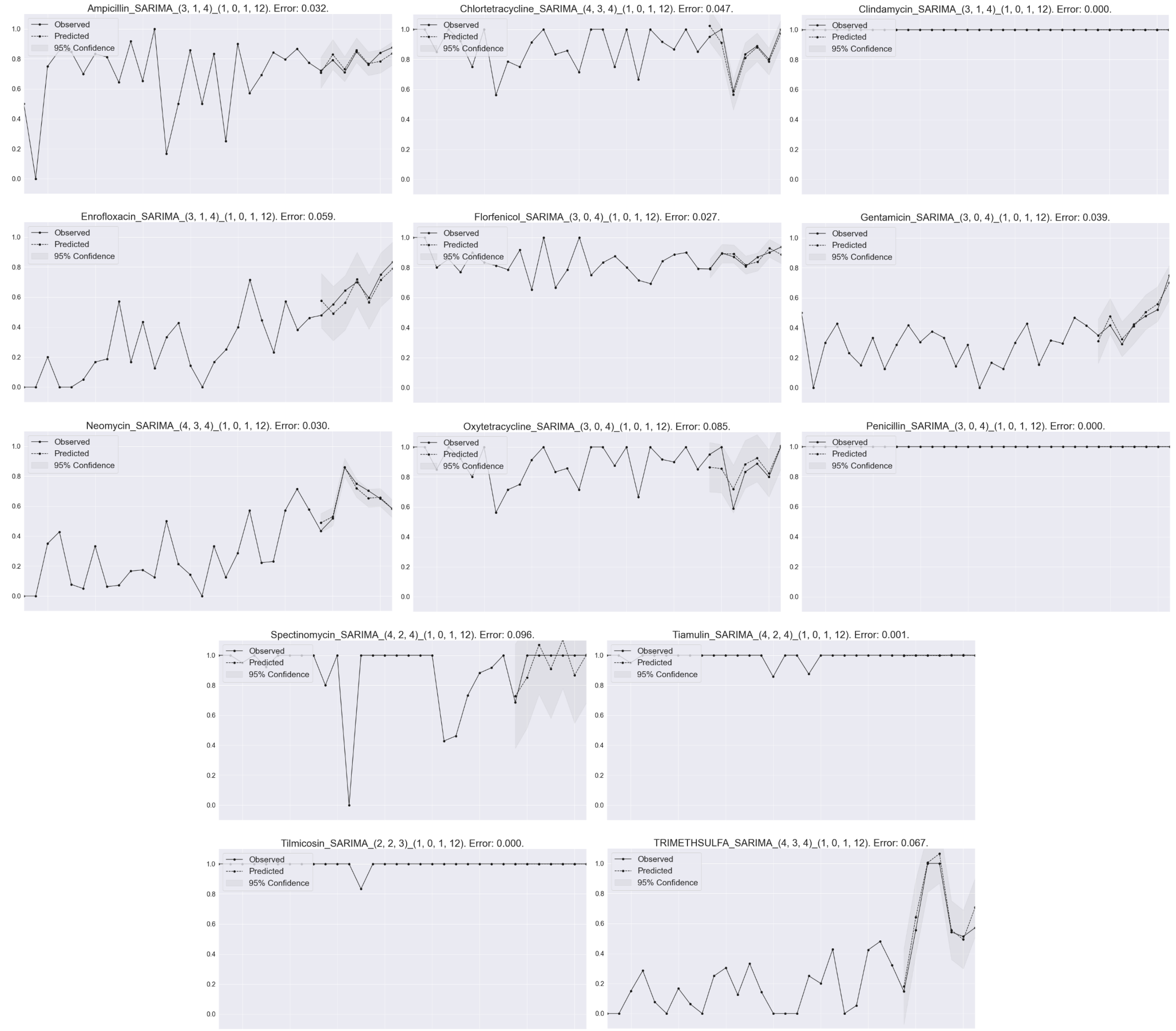

Supplementary 2. Prediction results on *Salmonella suis.* time series data samples based on the SARIMA (e.g. of 9 antibiotics). Solid and dotted lines are observed and predicted values, respectively, and the grey area is 95% prediction intervals.

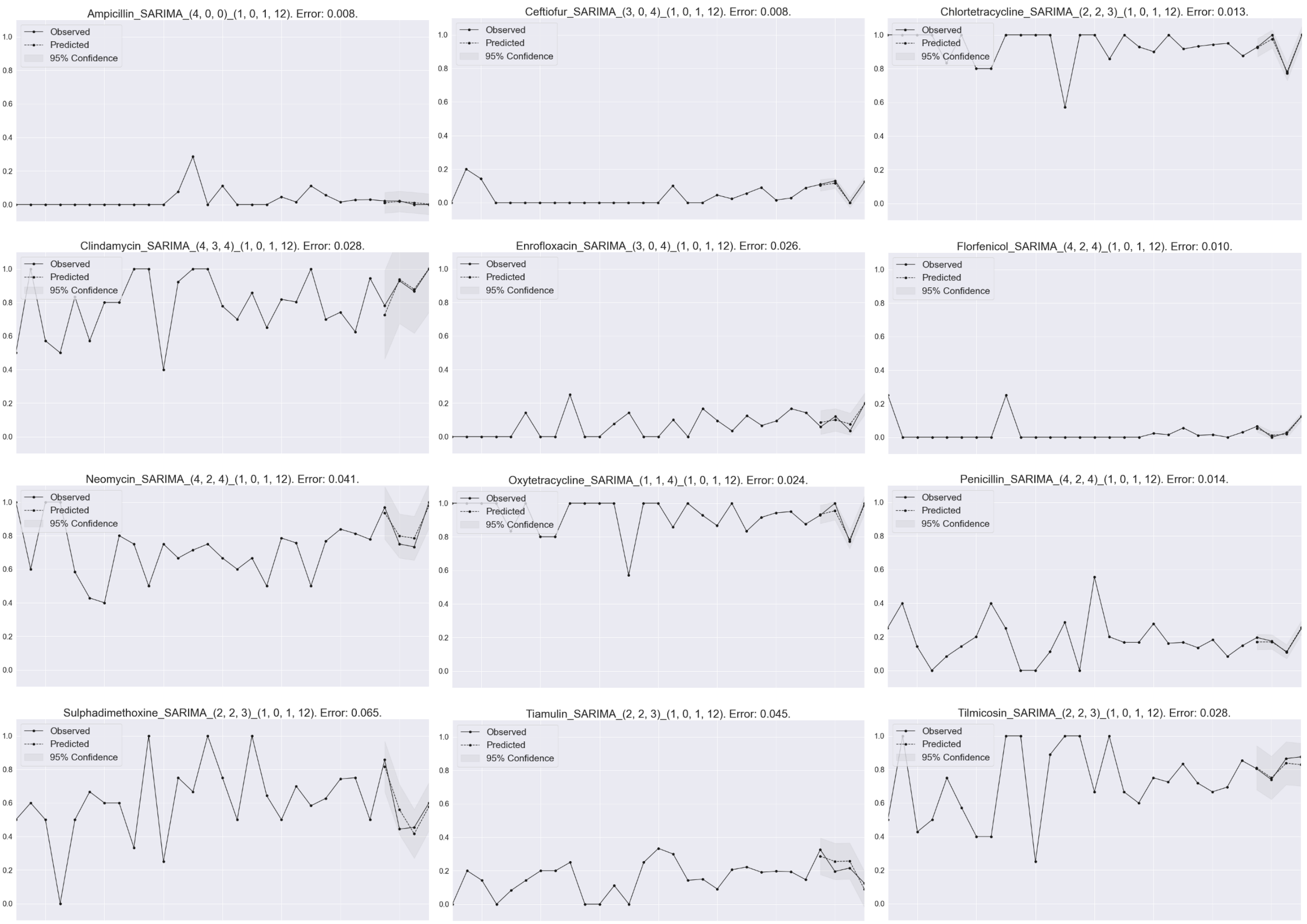

Supplementary 3. Prediction results on *Salmonella sp.* time series data samples based on the SARIMA (e.g. of 9 antibiotics). Solid and dotted lines are observed and predicted values, respectively, and the grey area is 95% prediction intervals.

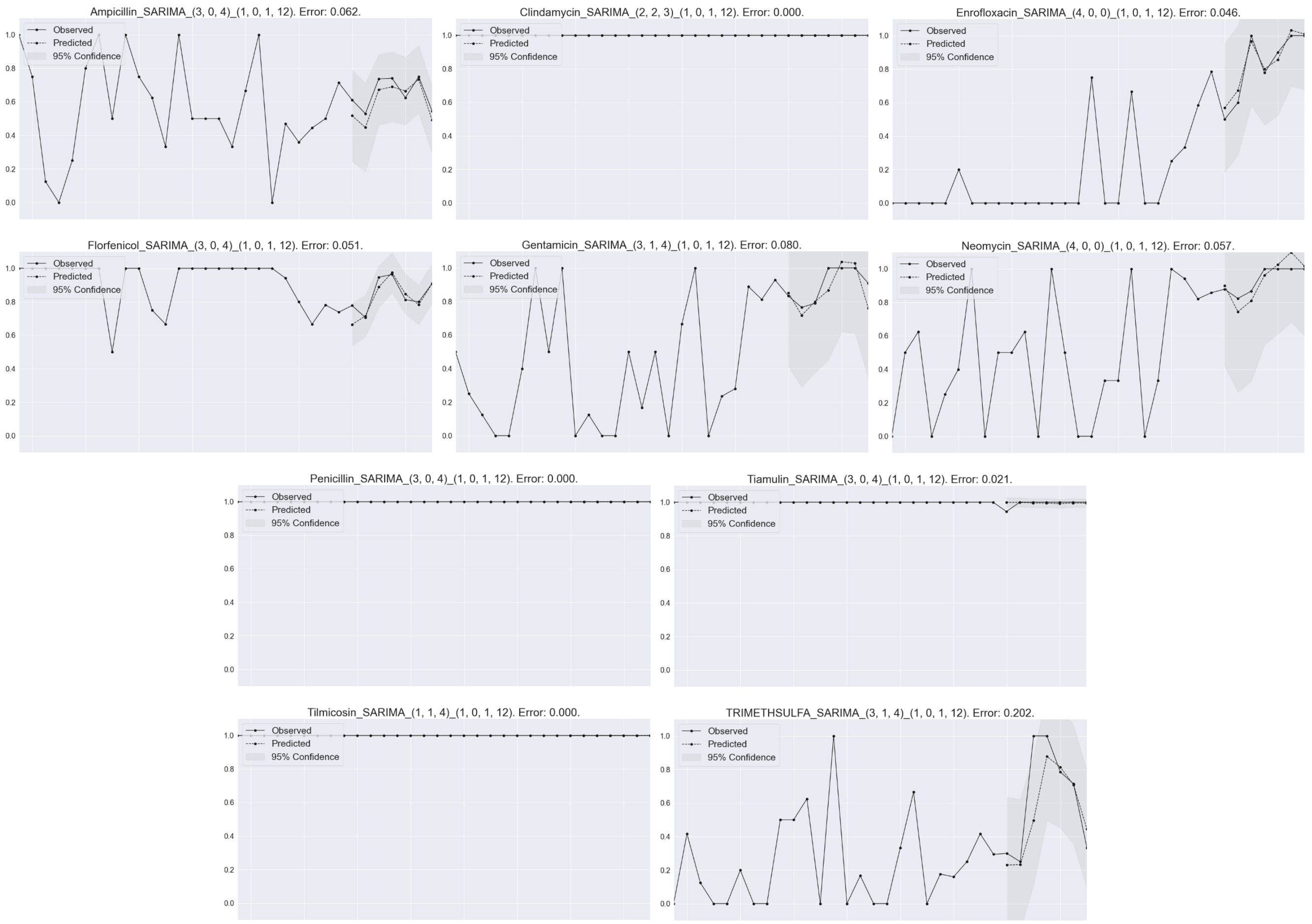

Supplementary 4. Prediction results on *Pasteurella multocida.* time series data samples based on the SARIMA (e.g. of 9 antibiotics). Solid and dotted lines are observed and predicted values, respectively, and the grey area is 95% prediction intervals.

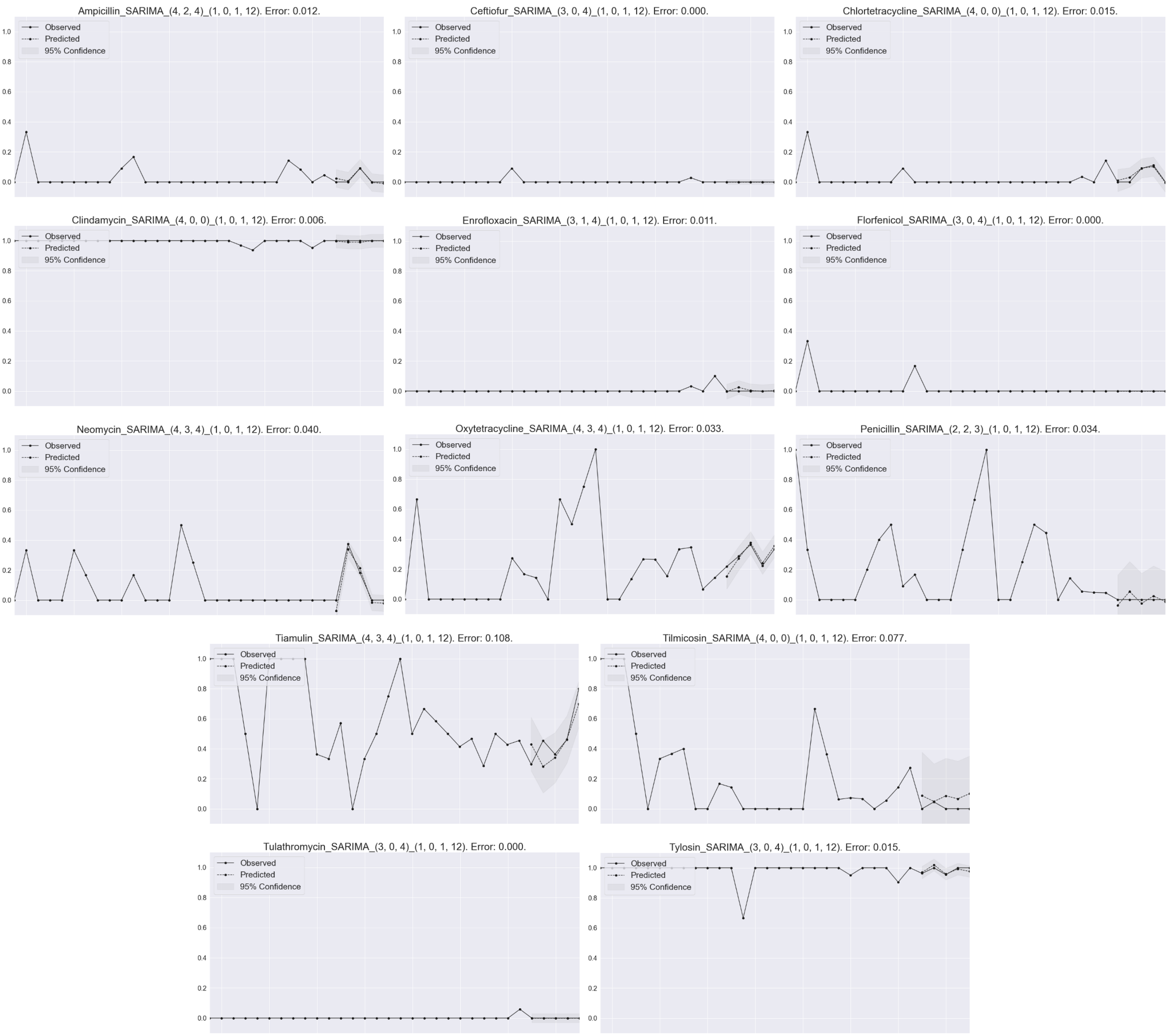

Supplementary 5. Prediction results on *B. bronchiseptica.* time series data samples based on the SARIMA (e.g. of 9 antibiotics). Solid and dotted lines are observed and predicted values, respectively, and the grey area is 95% prediction intervals.

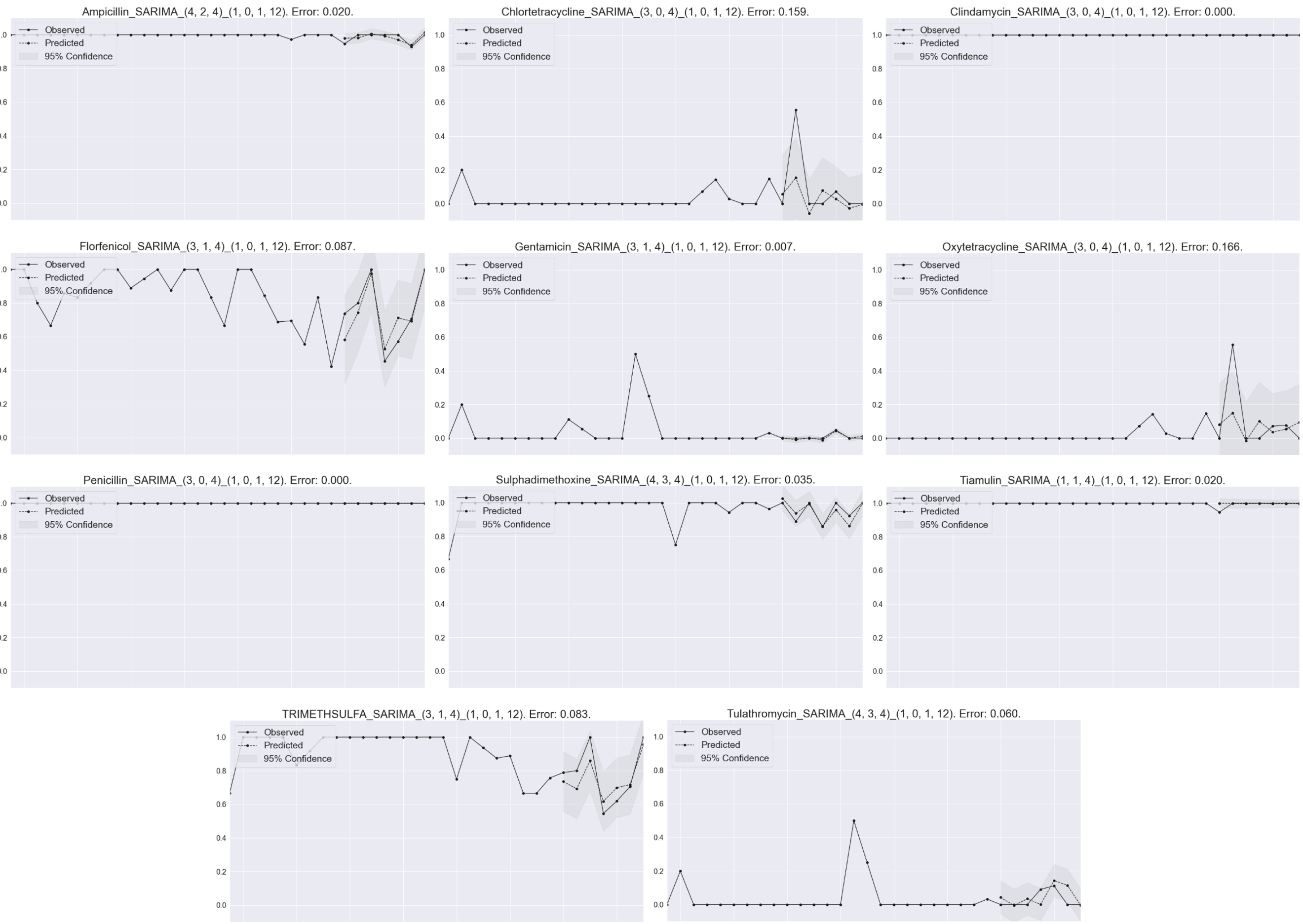

Supplement: Supplementary file 1 [file Data_Sheet_1.pdf]
